# A Systematic Review of the Bibliometrics and Methodological Research Used on Studies Focused on School Neighborhood Built Environment and the Physical Health of Children and Adolescents

**DOI:** 10.3390/children12070943

**Published:** 2025-07-17

**Authors:** Iris Díaz-Carrasco, Sergio Campos-Sánchez, Ana Queralt, Palma Chillón

**Affiliations:** 1 Department of Spatial Planning and Urban Design, School of Architecture, University of Granada, 18071 Granada, Spain; elcorreodeiris@ugr.es (I.D.-C.); scampos@ugr.es (S.C.-S.); 2 AFIPS Research Group, Department of Nursing, University of Valencia, 46010 Valencia, Spain; ana.queralt@uv.es; 3 Epidemiology and Environmental Health Joint Research Unit, FISABIO-UJI-UV, 46020 Valencia, Spain; 4 Department of Physical Education and Sports, Faculty of Sport Sciences, Sport and Health University Research Institute (iMUDS), University of Granada, 18011 Granada, Spain

**Keywords:** urban form, well-being, youth, teenagers, research design, method, educational center, device, buffer

## Abstract

**Objectives**: The aim of this systematic review is to analyze the research journals, sample characteristics and research methodology used in the studies about school neighborhood built environment (SNBE) and the physical health of children and adolescents. **Methods**: Using 124 key terms across four databases (Web of Science, PubMed, Sportdiscus and Transportation Research Board), 8837 studies were identified, and 55 were selected. The research question and evidence search were guided by the “Population, Intervention, Comparison, Outcomes” (PICO) framework. **Results**: Most studies were published in health-related research journals (67.3%) and conducted in 16 countries, primarily urban contexts (44.4%). Cross-sectional designs dominated (89.1%), with participation ranging from a minimum of 7 schools and 94 students to a maximum of 6362 schools and 979,119 students. Street network distances are often defined by 1000 or 800 m. The SNBE variables (135 total) were often measured via GIS (67.2%). In contrast, 70.6% of the 45 physical health measures relied on self-reports. **Conclusions**: This systematic review highlights the diverse approaches, gaps, and common patterns in studying the association between the SNBE and the physical health of children and adolescents. Therefore, this manuscript may serve as a valuable resource to examine the current landscape of knowledge and to guide future research on this topic.

## 1. Introduction

Global initiatives, such as the United Nations’ Sustainable Development Goals (SDGs) and the World Health Organization’s Healthy Cities Network, have highlighted the importance of collaborative efforts across departments and sectors to promote urban health, sustainability, and equity [1,2]. The built environment plays a crucial moderating role in shaping overall health outcomes [3] and contributes to the reduction of structural inequities—such as school segregation—through the implementation of well-designed and integrated policies [4]. Particularly, the association between the school neighborhood built environment (SNBE)—referring to the urban and architectural components surrounding the school—and the physical health (PH) of children and adolescents has emerged as a significant area of scientific research. There is evidence that the SNBE can directly impact adiposity [5], body mass index (BMI) [6,7], physical activity levels [8,9] or dietary choices [10,11].

Several interdisciplinary systematic reviews have been conducted to deepen our understanding of the methodologies used to study the built environment’s impact on health. These reviews have examined a range of tools for measurement, including audit instruments for evaluating green space quality in relation to mental health [12], observational tools for assessing food and physical activity (PA) environments in schools [13], and methodologies for measuring contextual exposure to the built environment in physical activity studies [14]. These systematic reviews have contributed to the development of a robust scientific framework for future research. Furthermore, it has been found that the choice of the study design can affect the results. For instance, in some cases, the study design significantly influenced the direction of the associations between the built environment and transit use [15]. Moreover, designing robust methodologies that can adequately capture the complex interactions between environmental factors and health outcomes is essential to obtain valid and reliable results.

However, there is currently no systematic review that compiles bibliometric data and research methodologies used to investigate the variables related to SNBE and PH in children and adolescents. For that reason, through this comprehensive analysis, the systematic review aims to shed light on the current landscape of research on SNBE and PH, providing insights that can facilitate improved decision-making for researchers and practitioners alike.

Given the interdisciplinary nature of the systematic review and considering the large number of approaches used to find associations between SNBE and PH among children and adolescents, the aims of this systematic review were (1) to identify which scientific journals have published articles investigating SNBE and PH and when they were published, (2) to identify sample characteristics and to report who the participants are in the identified studies, including their age group (children or adolescents) and where they come from, (3) to compile HOW research methodologies are employed in the identified studies, including the study design, the duration of data collection, the variables assessed along with their instruments, and the boundaries of the school area, and (4) to propose recommendations for addressing the findings and identified gaps, providing guidance for the design and implementation of future research studies.

## 2. Materials and Methods

Following a scientific recommendation [16] and methodologies applied in several systematic reviews focused on active commuting to and from school (ACS) [17,18,19], the journal articles were searched in the following four digital databases: (1) Web of Science Core Collection (the search was carried out in the Science Citation Index Expanded (SCI-EXPANDED) (1900–present), the Social Sciences Citation Index (SSCI) (1900–present), and the Arts & Humanities Citation Index (AHCI) (1975–present)), (2) PubMed, (3) Sportdiscus and (4) Transportation Research Board (TRB) library.

On scientific advice [20], the current systematic review has been adjusted to the Preferred Reporting Items for Systematic Reviews and Meta-Analyses Protocols (PRISMA-P) guidelines [20]. Likewise, the study protocol was registered in the PROSPERO International Prospective Register of Systematic Reviews, Supplementary S1 [21] (https://www.crd.york.ac.uk/prospero/ accessed on 14 July 2025), by X.X-X in March 2023 (CRD42023410860).

### 2.1. Search Strategy

The search terms focused on topics such as age, educational institutions, SNBE, and PH. These terms were combined through the use of various Boolean operators (“OR” and “AND”) to refine the search. Additionally, each search strategy was tailored to the specific characteristics of each database, covering the period from 1 January 2000 to 10 March 2023. As a result, searches were conducted in the title and abstract fields in PubMed, the title and/or abstract fields in Web of Science, the abstract field in SPORTDiscus, and based on full text in the Transportation Research Board (TRB) library. The “Population Intervention Comparison Outcomes” (PICO) strategy (Table 1) was used to frame the research question and the evidence search [22,23].

It should be noted that the selected keywords are based on previous systematic reviews on this topic [24,25,26,27,28,29,30,31,32,33,34,35,36,37].

### 2.2. Study Selection

**Figure 1:**
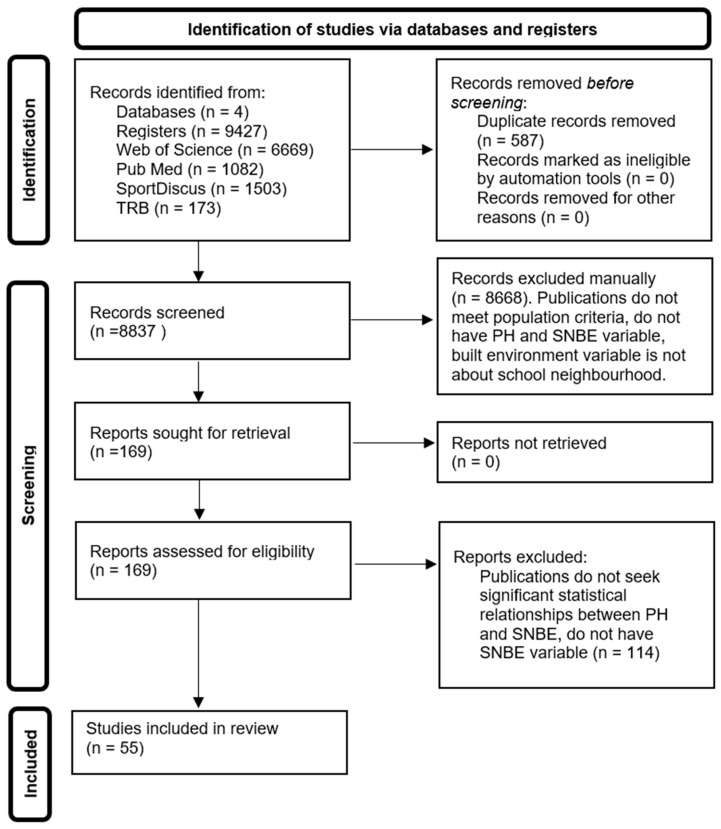
PRISMA flow diagram of the systematic review selection process.

The flowchart of the study selection process is presented in Figure 1. The initial search yielded 9427 articles, distributed as follows: 6669 from Web of Science, 1082 from PubMed, 1503 from SPORTDiscus, and 173 from TRB. After removing 590 duplicate entries using Zotero’s “Duplicate items” tool, 8837 unique articles remained.

Following standard recommendations, the final selection was conducted in three stages. First, titles and abstracts of the 8837 articles were independently screened by pairs (X.X-X with Y.Y and Z.Z.-Z), achieving a mean concordance of 97.31% (X.X-X with Y.Y) and 94.39% (X.X-X with Z.Z.-Z). In the second stage, 169 full-text articles were reviewed by the same pairs, with concordance rates of 82.85% (X.X-X with Y.Y) and 88.17% (X.X-X with Z.Z.-Z). After addressing any discrepancies, a full consensus was reached, with 100% agreement.

Ultimately, 55 articles were selected following this thorough screening process (Figure 1).

### 2.3. Data Extraction and Analysis

The information extracted from the selected studies was organized and summarized in Excel. For data manipulation, analysis, and visualization, a range of R packages within the RStudio 4.3.2 software were employed. Data import from Excel was facilitated using the foreign and readxl packages. To ensure data cleanliness and consistency, the janitor package was utilized for standardizing and correcting variable names. Core data manipulation tasks—such as filtering, selecting, and transforming variables—were conducted using the dplyr package. Additionally, stringr was employed for string processing and pattern matching operations, and tidyr was used for data tidying and reshaping. For statistical visualization and graphical representation of results, the ggplot2 package was the main package used. Word frequency and textual data were further explored using the wordcloud package. In terms of data export and reporting, the writexl and openxlsx packages facilitated the export of results to Excel format. Moreover, officer and flextable were used to generate formatted tables and documents suitable for presentation and publication. Finally, the DBI package was available to support database connections where necessary. RStudio (version 4.3.2) was selected instead of clustering-based tools [38] such as VOSviewer [39] due to its greater flexibility in data processing and visualization [40]. Since this study does not focus on thematic clustering, RStudio was more appropriate, as it enables researchers to tailor both the analytical and representational strategies to the specific characteristics of the dataset.

As shown in Supplementary S2, the extracted data for each study was organized into the following categories: year of publication, article title, journal title, continent, country, year (sample), sample size, percentage of girls, school sample, setting, school system, participant’s age, study design, PH variables, PH instrument, SNBE variables, SNBE instrument, street network distance to school and gender stratification.

## 3. Results

The findings of this systematic review are organized according to three primary thematic headings, aligned with the study’s objectives: (3.1) *Which and When: Mapping the Scientific Landscape Through Journals*; (3.2) *Where and Who: On-Site Portraits and Sample Characteristics*; and (3.3) *How: Research Pathways and Methodological Approaches*. These categories correspond to the key questions of the 5Ws framework—*Which*, *When*, *Where*, *Who*, and *How*—which structure the presentation of results below.

### 3.1. WHICH and WHEN: Mapping the Scientific Landscape Through Journals

**Figure 2:**
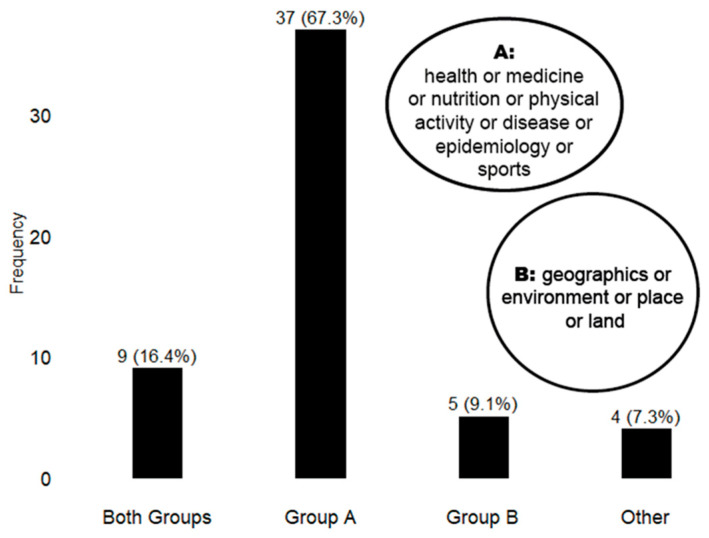
Academic journal classification of articles identified from the final screening.

Given the interdisciplinary nature of the systematic review, as the selected articles involve numerous disciplines such as urban planning, architecture, environmental science, sports science or nutrition, it is intriguing to explore the journal publication landscape (Figure 2). Notably, 67.53% of the articles were published in health-only journals, while 16.4% were featured in interdisciplinary journals covering both health and environment. Surprisingly, only 9.1% were published in journals exclusively dedicated to environmental studies.

**Figure 3:**
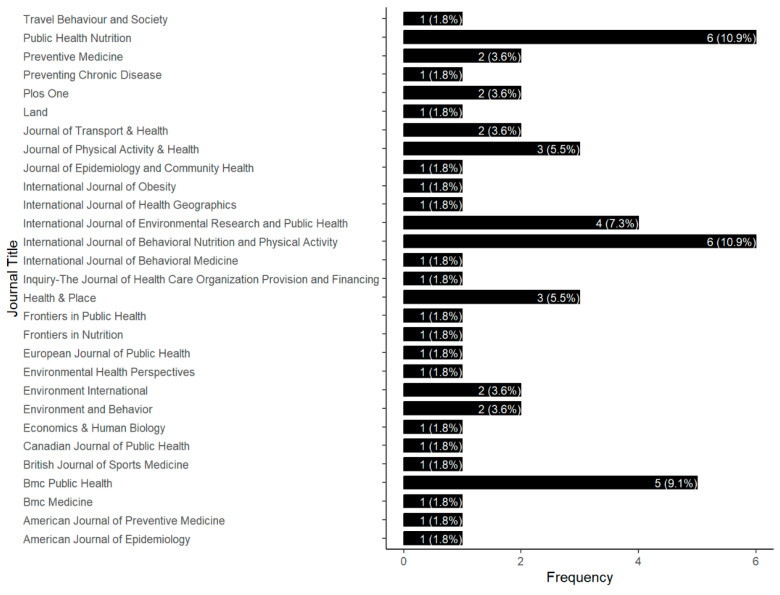
Journal title frequency.

The distribution of publications (Figure 3) showed a strong concentration, particularly in health-focused journals, with both the *International Journal of Behavioral Nutrition and Physical Activity* and *Public Health Nutrition* each comprising 10.9% of the total publications, followed closely by *BMC Public Health* with 9.1% frequency. Additionally, several academic journals that address both environmental and health issues were identified, including the *International Journal of Environmental Research and Public Health* (7.3%) and *Health & Place* (5.5%).

**Figure 4:**
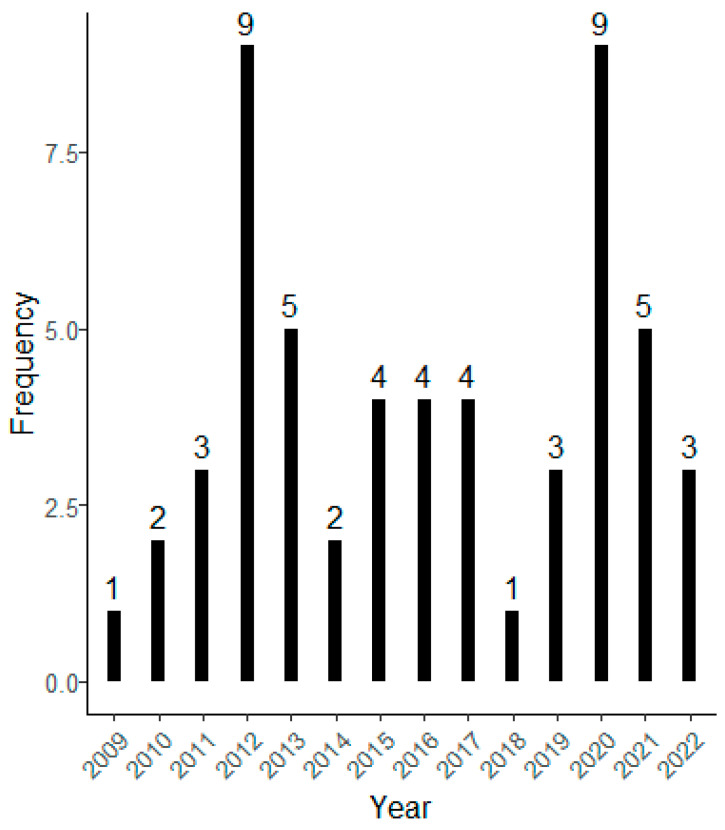
Distribution of articles according to the published year.

With regard to the frequency of publication over the years, it does not seem to be a consistent pattern (Figure 4). Specifically, in 2012 and 2020, nine articles were published each year. However, the years 2009 and 2018 saw only one publication each. The most frequently occurring number of publications per year is four, observed in 2015, 2016 and 2017.

### 3.2. WHERE and WHO: On-Site Portraits and Sample Characteristics

**Figure 5:**
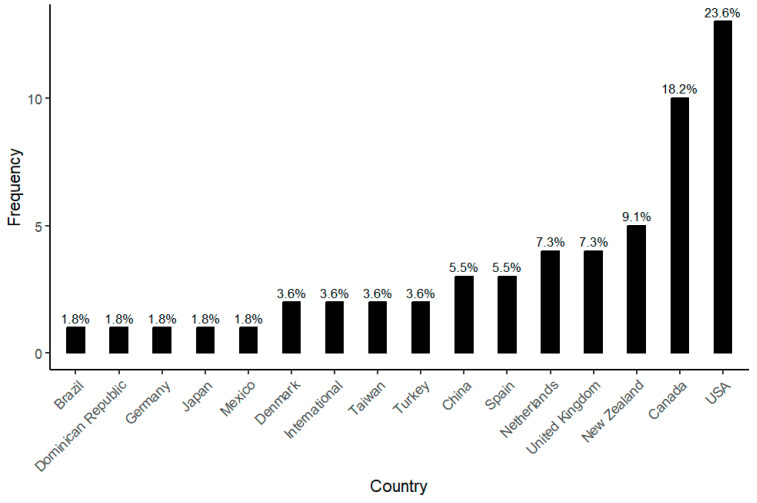
Country of origin of the studies based on participants’ data.

The geographical origin of the date of the identified studies is heterogeneous and occurs across continents (Supplementary S3 Figure S1): 47.2% of the studies come from America, with 25.5% from Europe, 14.4% from Asia, and 9.1% from Oceania, and 3.6% are intercontinental studies. Additionally, within the Americas, there are notable disparities, with 41.8% in North America, 3.6% in Central America, and 1.8% in South America. When considering the countries of origin (Figure 5), the leading contributors are the United States (23.6%) of America [41,42,43,44,45,46,47,48,49,50,51,52,53], followed closely by Canada with 18.2% of the articles [5,54,55,56,57,58,59,60,61,62], and the Dominican Republic [63], Mexico [9] and Brazil [64] with one article each (1.8%). In Oceania, New Zealand accounts for 9.1% [65,66,67,68,69], while in Europe, the Netherlands [70,71,72,73] and the United Kingdom [11,74,75,76] lead with 7.3% each, closely followed by Spain with 5.5% [77,78,79], Denmark with 3.6% [80,81] and Germany with 1.8% [82]. In Asia, China contributes 5.5% [83,84,85], closely followed by Taiwan [6,86] and Turkey [7,87], each with 1.8%, and Japan [9]. Finally, it should be noted that there is an absence of representation from Africa and Antarctica.

Considering the settings, the highest percentage is 44.4%, focused on the urban context. This result contrasts with the 3.7% of articles that have been conducted only in the rural context. It is noteworthy that there is a substantial percentage of unreported cases at 25.9%. This same percentage, 25.9%, has been reported in studies conducted across both urban and rural contexts (Supplementary S3 Figure S3).

**Figure 6:**
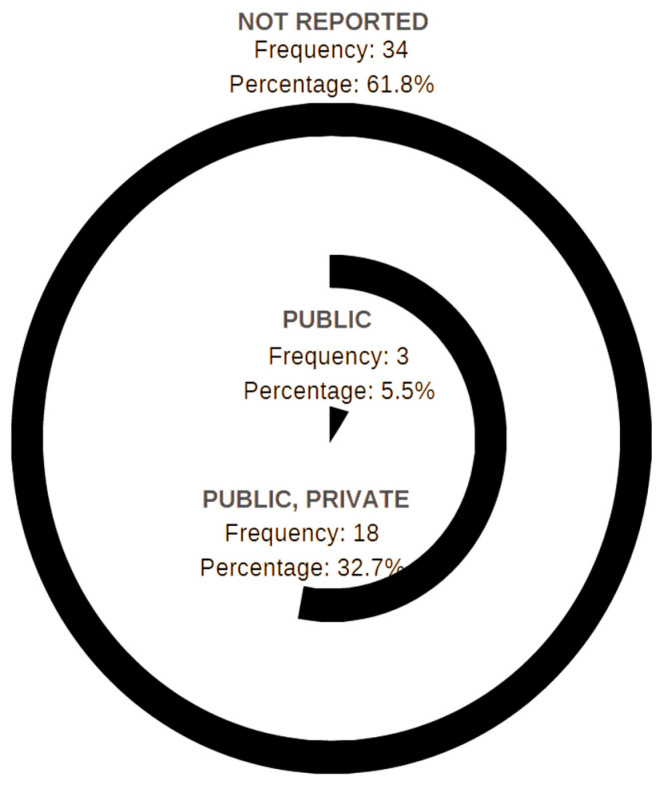
Descriptive statistics of the school systems reported in the selected articles.

Concerning the school system of the analyzed articles, it is noteworthy that 61.8% of them lack reporting on this aspect (Figure 6). Furthermore, 5.5% of the studies focus exclusively on public schools, while 32.7% examine both public and private institutions. Interestingly, none of the articles exclusively focus on private schools.

Regarding the analysis of school sample sizes (number of schools included), we observed a minimum of 7 schools and a maximum of 6362 schools. This wide range of values showed a significant difference between mean (268 schools) and median (46 schools). It should be noted that there is an 82.69% below-mean value. Likewise, of the 55 articles analyzed, 9 found no statistically significant association between SNBE and PH. Among these, eight reported school sample sizes, and of those, 88.9% (eight articles) had school sample sizes below the mean of 268 schools.

In terms of the number of individuals (children and adolescents) included in the analysis (sample size), a substantial range is evident, with the maximum and minimum sample sizes standing at 979,119 and 94, respectively, as shown in Table 2. Notably, there exists a considerable disparity between the mean sample size of 44,143.12 and the median sample size of 2304.5. It is worth mentioning that there is 90.74% below the mean value.

Within the articles studied, the peak percentage (45.5%) is observed in articles scrutinizing the health aspects of both adolescents and children. It is also worth mentioning that there exist more studies focusing only on children (36.4%) compared to those exclusively targeting adolescents (14.5%). Additionally, 3.6% of articles omit age specification (Supplementary S3 Figure S4). Regarding gender, the mean percentage of females is 49.2%. However, Similarly, the percentage of girls below the median is 48.93% (Supplementary S3 Figure S5). Likewise, eight articles did not specify the gender composition. Finally, it should be noted that only 14.4% of the articles analyze the associations between the SNBE and PH separately by gender.

### 3.3. HOW: Research Pathways and Methodological Approaches

Examining the study design, a distinct pattern emerges, with cross-sectional articles being the most prevalent (89.1%). Longitudinal studies constitute only a minority (7.3%), while there is a single instance of a case–control study (1.8%) and one observational article (1.8%) in residual numbers (Supplementary S3 Figure S2). There are no randomized controlled trials.

**Figure 7:**
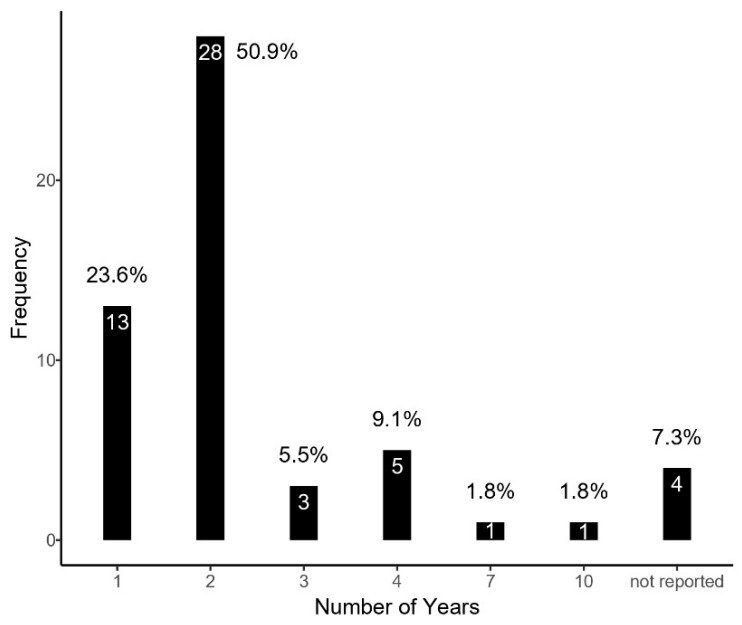
Duration of the data collection.

In terms of the duration of data collection, it is noteworthy that 50.9% of research teams allocated two years (Figure 7). Subsequently, 25.5% invested one year. Notably, only two articles extended their data collection period beyond five years.

**Figure 8:**
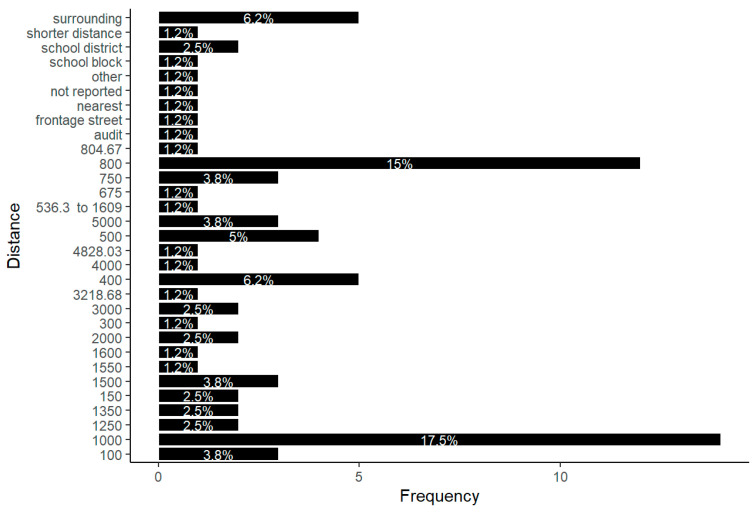
Chosen street network distance (length in meters) from the school for SNBE variable measurement.

The variables that have been studied in the systematic review and how they have been analyzed are set out below. On the one hand, the definition of SNBE requires clarification in order to define the boundaries of the school area. The identified articles use 31 different street network distances to define the school neighborhood area. As shown in Figure 8, 14 articles (17.5%) opted for a quantitative street network distance of 1000 m to define the SNBE. Following closely behind is 800 m, which was chosen by 12 articles (15%). Also, the street network distance to schools shows considerable variation, ranging from a minimum of 100 m to a maximum of 5000 m (three articles in both, 3.8%). It should be noted that about 28.12% of the observations fall below the median street network distance of 800 m. Conversely, a notable 87.5% of the data is below the mean street network distance of 1087.543 m. Regarding the non-numerical variables, ‘surrounding’ was the most common word used for defining school neighborhood (five articles, 6.2%).

**Figure 9:**
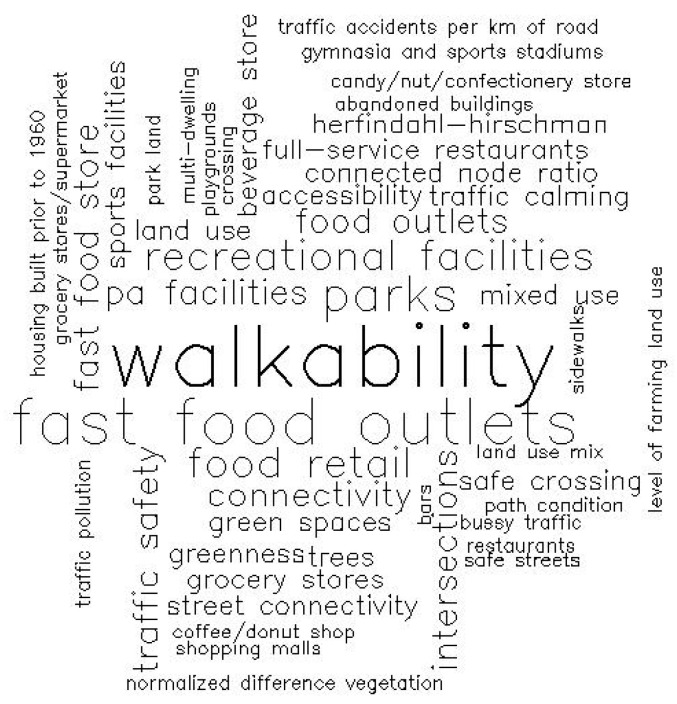
Variables used to analyze SNBE in the selected articles. The larger the size of the word in the figure, the more frequently it appears in the studies analyzed.

The following section analyzes which variables have been studied in this current systematic review. On the one hand, it reveals a wide range of SNBE variables (Figure 9) covering a total of 135 different variables. As shown in Supplementary S3 Figure S6, walkability stands out as the most frequently employed variable (7.4%), followed closely by convenience stores and fast-food restaurants (5.9%), fast food outlets (4.1%), and parks (3.4%). Additionally, land use mix and food retail (2.9% each) were used.

**Figure 10:**
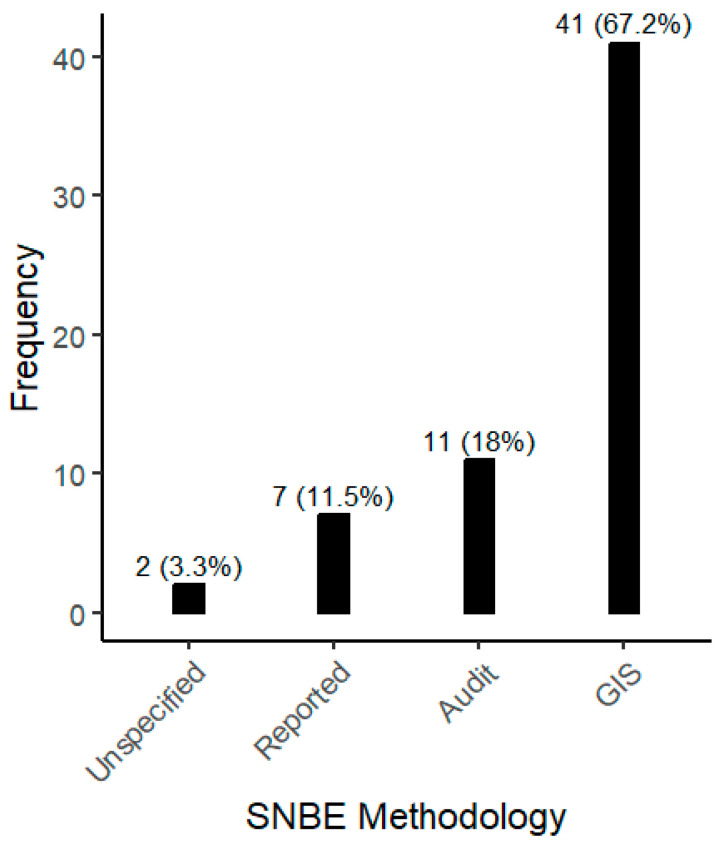
Methodology employed to analyze SNBE in the selected articles.

Concerning the methodologies used to evaluate SNBE characteristics, GIS emerges as prominent, being employed in 67.2% of the articles (Figure 10). Specifically, the software ArcGIS (Supplementary S3 Figure S8) was used in 29 articles, while QGIS was mentioned in 4. Additionally, other objective tools such as ArcView (11.3%) and alternative techniques including audit (18%) and self-reported methods (11.5%) were also used. It also should be mentioned that none of the audits used, e.g., MAPS or PEDS, are used more than once in the reviewed articles (Supplementary S3 Figure S8).

**Figure 11:**
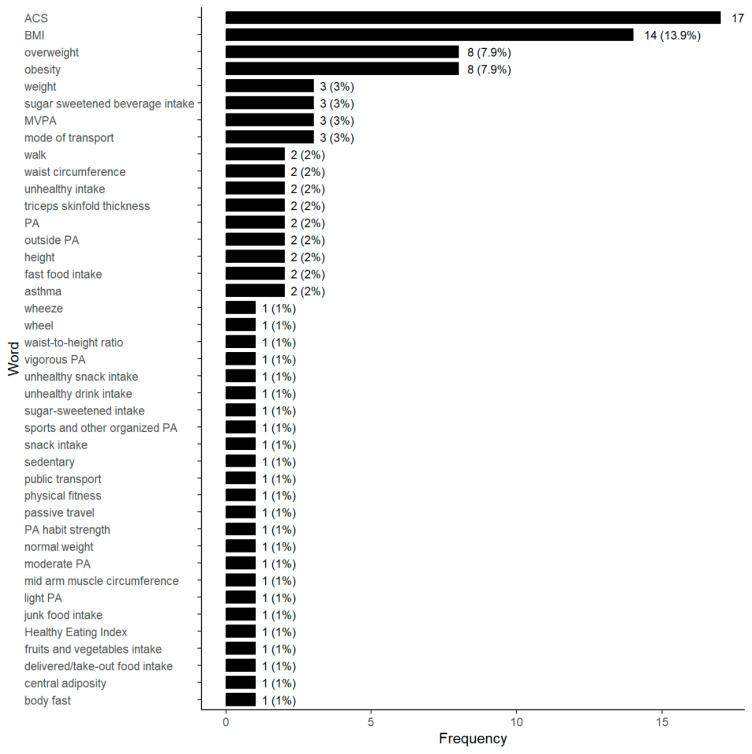
Type of PH variables in the selected studies.

When it comes to PH variables, our analysis reveals a more focused set, comprising only 45 distinct variables. In particular, ACS occurs 17 times (16.8%), while body mass index (BMI) is recorded 14 times (13.9%), highlighting their prevalence within the dataset (Figure 11). Furthermore, it is noteworthy that variables related to conditions such as obesity and overweight each appear eight times (7.9%), indicating their significant presence in the data. The remaining 41 variables appear three times, two times, or just once (1%).

**Figure 12:**
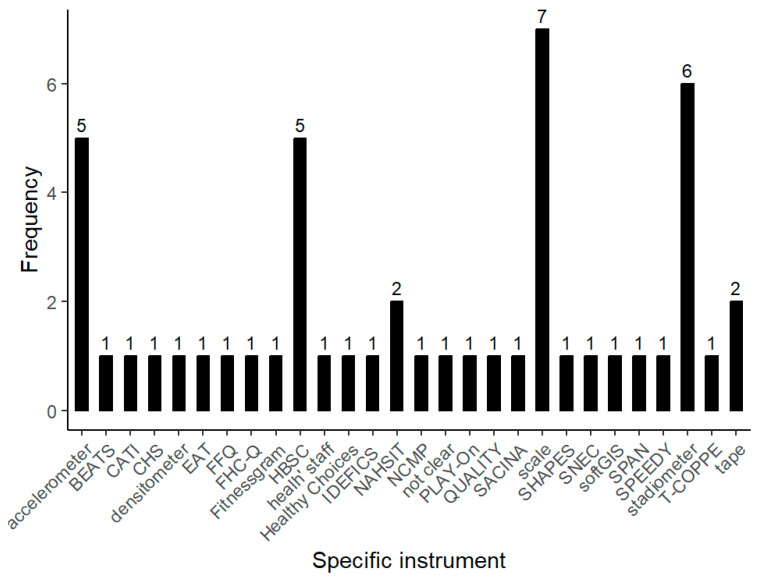
PH instruments used in the selected studies. Acronyms: BEATS (Built Environment and Active Transport to School), CATI (Computer-aided telephone interview), CHS (The Southern California Children’s Health Study), EAT (Eating and Activity in Teens), ESSENS (Environmental determinantS of dietary BehaviorS among adolescENtS), FFQ (Block Kids 2004), FHC-Q (Food, Health, and Choices questionnaire), HBSC (Canadian Health Behavior in School-Aged Children Survey), HC (Healthy Choices), IDEFICS (Identification and prevention of dietary- and lifestyle-induced health effects in children and infants), NAHSIT (Nutrition and Health Survey in Taiwan), NCMP (National Child Measurement Program), NEXT (Next Generation Health Study), QUALITY (Quebec Adipose and Lifestyle Investigation in Youth), SACINA (Self-Administered Children and Infants Nutrition Assessment), SHAPES (School Health Action, Planning, and Evaluation System), SPAN (School Physical Activity and Nutrition), SPEEDY (Sport and Physical activity and Eating behavior: Environmental determinants in Young people) and T-COPPE (Texas Childhood Obesity Prevention Policy Evaluation).

When it comes to the instruments used to assess PH indicators, a majority (70.6%) of the articles relied on self-reported questionnaires, while approximately 27.9% used objective measures (Supplementary S3 Figure S8). Among the objective instruments (Figure 12), scales and stadiometers were each mentioned in seven (14.3%) and six articles (12.2%), respectively, followed by accelerometers in five (10.2%). Densitometers and tape were sporadically mentioned (2%). In terms of surveys, the Canadian Health Behavior in School-Aged Children Survey (HBSC) was the sole recurring instrument, appearing six times (12.2%) throughout the analyzed articles. The other survey that is repeated is the Nutrition and Health Survey in Taiwan (NAHSIT), which appears twice (4.1%).

## 4. Discussion

The result confirms a certain homogeneity in the bibliometric data but also reveals highly heterogeneous methodological approaches.

The discussion is structured into two main sections: 4.1 (insights of the research: discussing the 5W), and 4.2 mentioning the strength, limitations, and future research. Section 4.1 focuses on a critical analysis of the research findings by addressing the five fundamental questions (Which, When, Where, Who and How), providing an in-depth understanding of the key trends, gaps, and patterns identified in the systematic review. Meanwhile, Section 4.2 highlights the strengths and limitations of the study, emphasizing the methodological rigor and areas where further research is needed. Additionally, it provides recommendations for future studies, aiming to address gaps in the current body of knowledge and guide the design of future research studies.

### 4.1. Insights of the Methodological Research: Discussing the 5W (Which, When, Where, Who and How)

The analysis presented in this systematic review (Figure 4), following the application of inclusion and exclusion criteria, did not reveal any evidence of exponential growth in the number of publications exploring the association between SNBE and the PH of children and adolescents. This is surprising given the growing interest in institutional initiatives like the STARS (Sustainable Travel Accreditation and Recognition for Schools) project [88]. Launched in 2013, the STARS initiative has been implemented in several countries, including the UK, Spain, Italy, Germany, and the Netherlands, among others. Its primary aim is to encourage a shift in student mobility habits toward more active, sustainable, and independent travel options. Today, the project is being developed in schools across nearly a dozen European cities. Another significant event has been the global COVID-19 pandemic. Furthermore, the global COVID-19 pandemic may represent a significant factor influencing the number of publications. This trend is evidenced by a scoping review, which highlighted an exponential increase in 2021, particularly in research focusing on built environments and its influence on COVID-19 [89]. The substantial rise in publications observed in 2020 within the framework of this systematic review may be attributed to the pandemic. Since its onset, restrictions on work and leisure activities have likely contributed to greater availability of time for both reviewers and authors, thereby fostering an increase in academic output.

Our findings reveal that most of the articles are published in health-related journals (particularly highlighting topics such as behavioral nutrition, physical activity and public health research). This finding may be reflected in our data analysis, as we observed a lack of focus on built environment considerations in some studies. For example, 25.9% of the articles do not report settings (urban and/or rural) and 61.8% do not specify the school system (public and/or private). This is surprising given that in many countries, such as Spain, access to public schools is based on home–school proximity, and specifically, proximity is one of the principles of chrono-urbanism [90]. The term chrono-urbanism emerged around 1997, influenced by Torsten Hägerstrand’s theoretical framework of time geography. This concept introduces a fresh perspective on urban planning, focusing on time as a critical resource within cities. Its core principle is that urban quality of life can be significantly enhanced by reducing the time residents spend traveling between various destinations [91]. Moreover, this review highlights examples of cities that have integrated chrono-urbanism [92] into their urban planning approaches, focusing on a time radius between 5 and 20 min. This aligns with the selection of street network distances used to calculate SNBE variables in the systematic review, as our findings show that 80.4% of the studies used distances ranging from 0.4 to 1.6 km, consistent with the range proposed in another review [93]. Curiously, among the articles that used GIS, the software ArcGIS (Supplementary S3 Figure S7) was employed in 87.87% of the studies. This is intriguing, as the software QGIS, unlike ArcGIS, is free software. This may be because ArcGIS has a specialized problem-solving tool that streamlines the process of addressing specific issues.

Regarding the geographical origin of the identified studies, there is a gap in research conducted in Africa and Antarctica; however, in North America, and particularly in the United States, there is a predominance of research on the associations between PH and SNBE. This trend is consistent with findings from another systematic review examining the associations between the physical environment and PA levels in preschool children [32]. Besides that, it is interesting to know that 44.4% of the studies are based on the urban context. This huge percentage is perhaps due to the importance of understanding the urban context, as urban areas in developing cities are expected to increase 2.5-fold in 2030 compared to 2000 [94]. Furthermore, it must be highlighted that defining urban and rural settings proved challenging due to the lack of standardized criteria. While numerous studies omit explicit definitions for these terms, a subset that is often classified employs population size as a defining factor. In particular, some studies [68] defined rural settings as areas with populations not exceeding 1000 inhabitants, whereas other research [95] extends the threshold to populations up to 20,000 inhabitants. Based on this premise, the definition of urban and rural areas may vary depending on national standards and geographic regions [96].

Additionally, we observed a lack of diversity in study design, particularly in longitudinal studies, case–control or pilot studies. This is confirmed by the fact that only two articles (3.6%) [5,48] extended their data collection period beyond five years. This may be due to the difficulty in obtaining data and long-term funding and/or the difficulty of interdepartmental collaborations.

In addition, the pattern that stands out in this systematic review is the substantial difference between the means and medians in both student samples (44,143.12 vs. 2304.5) and school samples (268 vs. 46). Regarding the size of the school sample, it is worth noting that of the 11 articles that found no statistical significant association between SNBE and PH, 8 (72%) had a school sample size below the mean, which may be due to insufficient statistical power [97]. The PH data’s wide-ranging diversity may be attributed to variations in healthcare infrastructure among countries. This might be attributed to certain countries benefiting from robust state health programs, facilitating extensive data collection, such as SHAPES in the USA [98] or HBSC in Canada [99].

Likewise, the systematic review observed that some articles targeted only children (36.4%) rather than adolescents (14.5%). This trend is intriguing considering that gathering data from adolescents is usually perceived as easier due to their autonomy in questionnaire responses or accelerometer wear. However, the emphasis on children could be attributed to their direct dependency on family members, making their involvement more feasible due to parental concerns for their well-being. Furthermore, differences are observed in the maximum sample size of students by sex. In some cases, these gender differences are important, as highlighted by a systematic review showing that the relationship between the built environment and time spent playing outdoors among boys and girls was different [100].

A total of 135 SNBE variables are documented. The walkability index emerges as the most frequently examined variable (7.4%). This interest in the walkability index can also be seen in the scientific literature worldwide, as there are several reviews that analyze its bibliometrics [101,102] or its measurement instruments [103,104]. After the walkability index, convenience store density, fast-food restaurant density (5.9% each) and fast-food outlet density (4.1%) are the most researched variables. This trend is also reflected in recent reviews, such as those on the influence of school neighborhood food environments on adolescent food purchases [105] and the links between convenience stores, fast-food restaurants and obesity in childhood [106,107] or in both children and adolescents [108]. Finally, it is worth mentioning park density (3.4%) as a topic of growing interest in scientific literature, particularly in studies investigating its connection to health. The importance of parks and their proximity to schools has been a focus of interest since the twentieth century. An example is Radburn’s design of the New York Regional Plan (1929), developed by C. Stein and H. Wright. Likewise, this trend is also reflected in several park-based intervention reviews [109,110]. When focusing on PH, in this systematic review, 45 unique variables were identified, with ACS and BMI being the most studied. This interest can be seen in reviews on weight status among adolescents [111] and active transportation among children [112,113].

It is interesting to highlight the opposite trends between SNBE and PH instruments, particularly the predominance of self-reported measures in studies evaluating the PH of adolescents. This means a notable lack of objective PH measures, since some scientific studies recommend the use of devices to measure PH, such as accelerometers and GPS sensors for ACS [114] or electronics scale and a telescopic height-measuring stick for assessing body composition [115].

### 4.2. Strengths, Limitations, and Future Research

The main strength of this study is that it is the first systematic review to comprehensively analyze the different approaches used to study the associations between the SNBE and the PH of children and adolescents.

For that reason, the article aims to serve as a reference for the design of studies investigating associations between the SNBE and PH. The research has other strengths, including compliance with PRISMA guidelines and the application of the PICO framework, supported by a comprehensive set of search terms (124 terms), a diverse range of databases (Web of Science, PubMed, SportDiscus, and the Transportation Research Board), and a rigorous screening process that reviewed 8837 articles over 22 years. Nevertheless, the limitations of this study lie in the article selection process, which included only manuscripts written in English and excluded studies published before the 21st century. Likewise, although this review focused exclusively on associations between SNBE and PH, it is important to note that these associations may be shaped by broader social environmental factors [116].

Following the analysis of research design gaps identified in this systematic review, we confirm the need for further studies exploring the associations between SNBE and PH. In this view, several recommendations are proposed to address these gaps, to promote the understanding of research design.

1. Regarding country representation, there are still 177 countries without a single published article investigating the associations between SNBE and PH. This finding underscores the need for broader geographic inclusivity to achieve a more comprehensive global perspective.
2. Due to the complex ecosystem of different variables (180) and in order to identify as exhaustively as possible the associations between SNBE and PH in adolescents and children, it is recommended to extend the project’s duration and to establish interdisciplinary research teams—bringing together experts in public health, urban design, education, and community engagement. Such collaborative efforts can significantly enhance the identification of relevant variables, data sources, and methodological approaches. Additionally, creating knowledge-sharing platforms, including online repositories and workshops, is advised to facilitate the exchange of findings, datasets, and methodological approaches, thereby promoting innovation and cross-disciplinary learning. Furthermore, academic institutions should be encouraged to develop integrated curricula that combine methodological training with discipline-specific knowledge and practical experiences in urban settings to better prepare future researchers to understand the complex impact of the built environment on health outcomes. Finally, promoting interdisciplinary collaboration may also contribute to a more balanced representation of research in the scientific literature, particularly through increased publication in interdisciplinary journals. Such efforts can facilitate integrative frameworks, break down disciplinary silos, and advance a more comprehensive understanding of the associations between SNBE and physical health outcomes.
3. It is recommended to establish a clear definition of urban and rural areas in the context of SNBE, given the variety of standardized definitions currently in use. It is also suggested to increase the number of studies comparing urban and rural contexts to establish similarities and differences, since only 25.9% of the articles have studied both contexts.
4. It is suggested to increase the number of longitudinal research studies and case–control studies, as these remain notably underrepresented. Longitudinal designs are essential for establishing temporal sequences and inferring potential causal relationships, thereby offering more robust evidence on the long-term impact.
5. It is important to report on the type of school system, as it may influence both the characteristics of the SNBE and/or adolescent travel behaviors. In many countries, access to public schools is determined by home–school proximity, which is a core principle of chrono-urbanism [90]. Therefore, analyzing and comparing the SNBE of public and private schools is recommended, as potential differences in their surrounding contexts may have implications for the PH of children and adolescents. This approach is especially pertinent in light of established associations between residential and educational segregation [117,118].
6. To facilitate cross-study comparability, it is advisable to employ buffer distances of 800 m and 1000 m, as these are the most commonly standardized distances.
7. It would be valuable to explore more scientific literature with a high representation of girls as well as to analyze participants separately to discern potential variations in results based on gender.
8. It is advisable to increase the use of device-based measurements in both SNBE and PH dimensions to improve the accuracy of the results.
9. It is recommended to analyze the differences between the use of ArcGIS and QGIS software in analyzing the built environment to determine whether the prominence of ArcGIS is due to its superior capabilities or other factors.

## 5. Conclusions

After reviewing and analyzing 55 articles, the bibliometric data indicate that the field is predominantly shaped by research conducted in North American contexts and published in health-focused journals. Regarding the methodology employed, most studies are cross-sectional and rely heavily on self-reported health measures, with limited use of objective indicators. Significant variations exist in the number of schools selected, the types of schools chosen, the number of participants, the methods used to collect PH variables, the SNBE variables analyzed, and the street network distances used to define the SNBE. Despite these differences, we observed some consistency in the variables chosen to assess PH.

In conclusion, this systematic review highlights the diverse approaches, existing gaps, and common patterns in studying the association between SNBE and the PH of children and adolescents. The review reveals a fragmented evidence base, underscores the need for methodological standardization, and provides recommendations to address current research gaps. This comprehensive overview of the research landscape may serve as a valuable tool to guide the design and implementation of future studies.

## Figures and Tables

**Table 1:** PICO strategy: category, definition, and search terms.

Category	Definition	Search Terms
Population	Individuals between 3 and 18 years old who were enrolled in kindergarten, primary school or high school.	**Age:** children* OR adolescen* OR youth* OR young* OR juven* OR teen* OR student* OR pupil* OR infan* OR boy* OR girl* OR kid* OR child* OR pediatric* OR schoolchildren* OR puber* OR minor* OR preschooler* **Education center**: school* OR institute* OR preschool* OR kindergarden* OR kindergarten*
Interventions	Scientific articles in English, using both urban planning and design variables	**School neighboorhood built environment (SNBE)**: “urban renewal*” OR forest* OR “traffic calming” OR “traffic light*” OR buil* OR “recreation* facility” OR park* OR garden* OR “sport* field*” OR “basketball field*” OR “soccer* field*” OR “playground*” OR green* OR space* OR “environment* correlates” OR “physical environment” OR “physical activ* environment” OR “food environment*” OR “environment* condition*” OR “environment* plan” OR “environment* design*” OR “urban* form*” OR “urban* service*” OR “urban* plan*” OR “urban* design*” OR “cit* plan*” OR architectur* OR streetscape* OR “land use*” OR crosswalk* OR sidewalk* OR pavement* OR “aesthetic* qualit*” OR densit* OR road* OR street* OR route* OR trail* OR highway* OR freeway* OR footway* OR walkway* OR footpath* OR infrastructure* OR amenit* OR path* OR “physical barrier*” OR slope* OR “setting intervention*” OR block* OR district* OR neighbourhood* OR neighborhood* OR “census block*” OR “census tract*”
Comparisons	Not applicable	Not applicable
Outcomes	Scientific articles whose results are obtained from the association of at least one SNBE variable with at least one or more PH variable. The dimensions of PH are physical activity, physical fitness, dietary intake and nutrition, cardiometabolic factors and respiratory health	**Physical Health (PH):** health* OR fit* OR “physical wellbeing” OR weight* OR overweight* OR obesity* OR weight* OR obesity OR “cardiorespiratory endurance” OR “muscular endurance” OR “muscular strength” OR “body composition” OR flexibility OR respirat* OR breath* OR “bod* mass index” OR “blood* profile*” OR BMI OR “waist circumference” OR nutrition* OR eat* OR diet* OR food* OR walk* OR step* OR commut* OR stroll* OR travel* OR transport* OR displacement* OR run* OR cycli* OR rid* OR activ* OR exercis* OR sport* OR “physical exertion” OR “physical activity”

**Table 2:** Descriptive picture of the sample size from the selected studies.

	Student Sample (n°)	School Sample (n°)	Female (%)
Maximum	979,119	6362	59
Minimum	94	7	25
Mean	44,143	268	49
Median	2304	46	50
Percentage Below Median	50	50	66
Percentage Below Mean	91	83	34

## Data Availability

All the data of this study are provided in the Supplementary Materials.

## Supplementary Materials

The following supporting information can be downloaded at https://www.mdpi.com/article/10.3390/children12070943/s1: Supplementary S1: PROSPERO International Prospective Register of Systematic Reviews; Supplementary S2: Extracted data from the systematic review; Supplementary S3: Figure S1: Continents; Figure S2: Study design; Figure S3: Setting; Figure S4: Participants’ age; Figure S5: Female participants; Figure S6: SNBE variables; Figure S7: SNBE-specific instruments; Figure S8: Instruments (PH).

## Abbreviations

The following abbreviations are used in this manuscript:

ACS	Active commuting to and from (to/from) school
ASPEN	Assessment System for Population Exposure Nationwide
BEATS	Built Environment and Active Transport to School
BMI	Body Mass Index
Can-ALE	Intersection density, dwelling density, and points of interest
CATI	Computer-aided telephone interview
CHS	The Southern California Children’s Health Study
EAT	Eating and Activity in Teens
ESATS	Environmental Scan for Active Transport to School
ESSENS	Environmental determinantS of dietary BehaviorS among adolescENtS
FFQ	Block Kids 2004
fhc-q	Food, Health, and Choices questionnaire
FSR	Full-Service Restaurants
FVI	Fruit and vegetable intake
GIS	Geographic Information System
HBSC	Canadian Health Behavior in School-Aged Children Survey
HC	Healthy Choices
HEI	Healthy Eating Index
HHI	Herfindahl–Hirschman index
IDEFICS	Identification and prevention of dietary- and lifestyle-induced health effects in children and infants
IMI	The Irvine Minnesota Inventory
MAPS	Microscale Audit Pedestrian Streetscapes MAPS-Global audit tool
MPA	Moderate Physical Activity
MVPA	Moderate to Vigorous Physical Activity
NAHSIT	Nutrition and Health Survey in Taiwan
NCMP	National Child Measurement Programme
NDVI	Normalized Difference Vegetation Index
NEXT	Next Generation Health Study
PA	Physical activity
PEDS	Pedestrian Environment Data Scan
PH	Physical Health
QUALITY	Quebec Adipose and Lifestyle Investigation in Youth
SACINA	Self-Administered Children and Infants Nutrition Assessment
SHAPES	School Health Action, Planning, and Evaluation System
SNBE	School Neighborhood Built Environment
SNEC	Seven Northeast Cities Study
SOPA	Sports and other organized physical activities
SPAN	School Physical Activity and Nutrition
SPEEDY	Sport and Physical activity and Eating behaviour: Environmental determinants in Young people
T-COPPE	Texas Childhood Obesity Prevention Policy Evaluation
VPA	Vigorous Physical Activity
