# Supplementary material for: A Systematic Review of the Bibliometrics and Methodological Research Used on Studies Focused on School Neighborhood Built Environment and the Physical Health of Children and Adolescents
Source: Children (Basel). 2025 Jul 17;12(7):943. doi: 10.3390/children12070943 (PMC12293611; doi:10.3390/children12070943)
Supplement: Supplementary file 1 [file children-12-00943-s001.zip › Supplementary S1.pdf]

Systematic review

A list of fields that can be edited in an update can be found here

1. * Review title.

Give the title of the review in English

"Exploring the relationship between school neighbourhood physical environment and physical health in children and adolescents: a systematic review"

2. Original language title.

For reviews in languages other than English, give the title in the original language. This will be displayed with the English language title.

3. * Anticipated or actual start date.

Give the date the systematic review started or is expected to start.

03/10/2022

4. * Anticipated completion date.

Give the date by which the review is expected to be completed.

15/05/2023

5. * Stage of review at time of this submission.

This field uses answers to initial screening questions. It cannot be edited until after registration.

Tick the boxes to show which review tasks have been started and which have been completed.

Update this field each time any amendments are made to a published record.

The review has not yet started: No

Review stage	Started	Completed
Preliminary searches	Yes	Yes
Piloting of the study selection process	Yes	Yes
Formal screening of search results against eligibility criteria	Yes	No
Data extraction	No	No
Risk of bias (quality) assessment	No	No
Data analysis	No	No

Provide any other relevant information about the stage of the review here.

6. * Named contact.

The named contact is the guarantor for the accuracy of the information in the register record. This may be any member of the review team.

Iris Díaz-Carrasco

Email salutation (e.g. "Dr Smith" or "Joanne") for correspondence:

Díaz-Carrasco

7. * Named contact email.

Give the electronic email address of the named contact.

irisdc524@gmail.com

8. Named contact address

Give the full institutional/organisational postal address for the named contact.

9. Named contact phone number.

Give the telephone number for the named contact, including international dialling code.

+34677098567

10. * Organisational affiliation of the review.

Full title of the organisational affiliations for this review and website address if available. This field may be completed as 'None' if the review is not affiliated to any organisation.

-Department of Spatial Planing and Urban Design of the University of Granada. RNM-357: Urban Laboratory

-Department of Physical Education and Sports, Faculty of Sport Sciences, Sport and Health University Research Institute (iMUDS), University of Granada, Granada, Spain

Organisation web address:

https://profith.ugr.es/ [illegible]

11. * Review team members and their organisational affiliations.

Give the personal details and the organisational affiliations of each member of the review team. Affiliation refers to groups or organisations to which review team members belong. **NOTE: email and country now MUST be entered for each person, unless you are amending a published record.**

Iris Díaz-Carrasco. Granada University
Palma Chillón-Garzón. Department of Physical Education and Sports, Faculty of Sport Sciences, Sport and Health University Research Institute (iMUDS), University of Granada, Granada, Spain
Sergio Campos-Sánchez. Department of Spatial Planing and Urban Design of the University of Granada. RNM-357: Urban laboratory

12. * Funding sources/sponsors.

Details of the individuals, organizations, groups, companies or other legal entities who have funded or sponsored the review.

This study was supported by the Spanish Ministry of Economy, Industry and Competitiveness and the European Regional Development Fund (DEP2016-75598-R, MINECO/FEDER, UE), and Spanish Ministry of Education and Vocational Training (FPU17/03934 and FPU18/04521).

Additionally, the research was funded by FEDER/Junta de Andalucía - Ministry of Economic Transformation, Industry, Knowledge and Universities (Reference B-CTS-160-UGR20).

Grant number(s)

State the funder, grant or award number and the date of award

Iris Díaz-Carrasco is supported by a research project contract from the University of Granada, charge to CiudActiva project: Influence of the urban environment on the physical activity behaviors of Andalusian adolescents, B-CTS-160-UGR20, R&D Projects of the FEDER 2020 Operational Program, published on 09/12/2022, Annex 27, Ref .225.

13. * Conflicts of interest.

List actual or perceived conflicts of interest (financial or academic).

None

14. Collaborators.

Give the name and affiliation of any individuals or organisations who are working on the review but who are not listed as review team members. **NOTE: email and country must be completed for each person, unless you are amending a published record.**

15. * Review question.

State the review question(s) clearly and precisely. It may be appropriate to break very broad questions down into a series of related more specific questions. Questions may be framed or refined using PI(E)COS or similar where relevant.

¿Is there any relationships between school neighbourhood physical environment and children and/or adolescents physical health?

16. * Searches.

State the sources that will be searched (e.g. Medline). Give the search dates, and any restrictions (e.g. language or publication date). Do NOT enter the full search strategy (it may be provided as a link or attachment below.)

Web of Science Core Collection: The search was carried out in the editions of Science Citation Index Expanded (SCI-EXPANDED) 1900-present, Social Sciences Citation Index (SSCI)-1900-present and Arts & Humanities Citation Index(AHCI) 1975-present, (2) PubMed, (3) SPORTDiscus and (4)Transportation Research Board TRB library.

-Publications years up to 2020

-Only studies in English

-Protocol studies, reviews, editorials, and abstract or congress communications were excluded

17. URL to search strategy.

Upload a file with your search strategy, or an example of a search strategy for a specific database, (including the keywords) in pdf or word format. In doing so you are consenting to the file being made publicly accessible. Or provide a URL or link to the strategy. Do NOT provide links to your search **results**.

Alternatively, upload your search strategy to CRD in pdf format. Please note that by doing so you are consenting to the file being made publicly accessible.

Do not make this file publicly available until the review is complete

18. * Condition or domain being studied.

Give a short description of the disease, condition or healthcare domain being studied in your systematic review.

Physical health: physical activity, physical fitness, dietary intake and nutrition, cardiometabolic factors and respiratory factors.

19. * Participants/population.

Specify the participants or populations being studied in the review. The preferred format includes details of both inclusion and exclusion criteria.

Individuals between three and 18 years old. Participants in the included studies were enrolled in kindergarten, primary school or high school in low, middle or high income countries.

20. * Intervention(s), exposure(s).

Give full and clear descriptions or definitions of the interventions or the exposures to be reviewed. The preferred format includes details of both inclusion and exclusion criteria.

The physical environment refers to the natural or built surroundings in which living organisms, including humans, exist. It includes the air, water, soil, climate, landforms, and other physical features of the earth. The physical environment can also refer to the human-made or built environment, including buildings, roads, infrastructure, and other constructed features.

The dimensions of physical health are: physical activity, physical fitness, dietary intake and nutrition, cardiometabolic factors and respiratory health.

21. * Comparator(s)/control.

Where relevant, give details of the alternatives against which the intervention/exposure will be compared (e.g. another intervention or a non-exposed control group). The preferred format includes details of both inclusion and exclusion criteria.

Not applicable

22. * Types of study to be included.

Give details of the study designs (e.g. RCT) that are eligible for inclusion in the review. The preferred format includes both inclusion and exclusion criteria. If there are no restrictions on the types of study, this should be

stated.

Published in English in a peer-reviewed journal: cross-sectional, longitudinal, and interventional designs (i.e., randomised trials (i.e., cluster randomised trials) and non-randomised studies (e.g., quasi-experimental studies, matched studies, non-matched studies, single group, and pilot studies)) accessible in their entirety.

23. Context.

Give summary details of the setting or other relevant characteristics, which help define the inclusion or exclusion criteria.

24. * Main outcome(s).

Give the pre-specified main (most important) outcomes of the review, including details of how the outcome is defined and measured and when these measurement are made, if these are part of the review inclusion criteria.

To compile and to review which variables are analysed in the school neighbourhood physical environment and there are linked to physical health

Measures of effect

Please specify the effect measure(s) for you main outcome(s) e.g. relative risks, odds ratios, risk difference, and/or 'number needed to treat.

25. * Additional outcome(s).

List the pre-specified additional outcomes of the review, with a similar level of detail to that required for main outcomes. Where there are no additional outcomes please state 'None' or 'Not applicable' as appropriate to the review

We will propose practical recommendations to assess healthy school neighbourhood physical environment based on the studies identified

Measures of effect

Please specify the effect measure(s) for you additional outcome(s) e.g. relative risks, odds ratios, risk difference, and/or 'number needed to treat.

26. * Data extraction (selection and coding).

Describe how studies will be selected for inclusion. State what data will be extracted or obtained. State how this will be done and recorded.

The articles were simultaneously selected by three reviewers (I.D-C, P.C-G and S.C-S). As recommended in the literature, the selection has been carried out in three steps (Gunnell et al., 2020). In the first step, , titles and abstracts were screened, and, where there was doubt about their inclusion, the full text was reviewed. In the event of disagreement or mismatch between the three main researchers, the opinion of the majority, i.e. the common opinion of two of them, was the one taken into account. In the second step, full-text articles of

eligible studies were reviewed for inclusion. In the third step, the references of the selected articles were carefully analysed to identify any other articles that could have been ignored in our search strategy. In addition, we analysed the references of systematic reviews similar to our topic, in order to identify any other articles that meet our inclusion criteria.

The "Duplicate items" tool of the Zotero bibliographic manager was used to remove duplicate references.

27. * Risk of bias (quality) assessment.

State which characteristics of the studies will be assessed and/or any formal risk of bias/quality assessment tools that will be used.

Following the recommendations of a recent systematic review (Campos-Garzón et al., 2020; Gunnell et al., 2020), both the risk of bias and the quality of the identified studies will be evaluated.

Evaluation of risk of bias of each included study were done with Cochrane Methods: (a) Randomized trials were evaluated with “RoB 2 tool” (Sterne et al., 2019), (b) non-randomised studies were evaluated with ‘’ROBINS-I tool” (“ROBINS-I: a tool for assessing risk of bias in non-randomised studies of interventions | The BMJ,” n.d.) and (c) observational studies were analysed with an adaptation of ‘’The Cochrane Collaboration’s Tool for Assessing Risk of Bias(Poitras et al., 2016; Prince et al., 2017).

Secondly, in order to assess the degree of confidence in included studies, quality assessment was done using Quality Assessment Tool for Observational Cohort and Cross-Sectional Studies and Quality Assessment Tool of Controlled Intervention Studies.

28. * Strategy for data synthesis.

Describe the methods you plan to use to synthesise data. This **must not be generic text** but should be **specific to your review** and describe how the proposed approach will be applied to your data. If meta-analysis is planned, describe the models to be used, methods to explore statistical heterogeneity, and software package to be used.

According to previous research (Schönbach et al., 2019), depending on the findings, the authors will decide to carry out a meta-analysis or a narrative synthesis.

Data were extracted independently using standardized electronic templates (Excel, Microsoft 365). The following research findings were recorded from each selected study in a summary table s: (a) first author; (b) year ; (c) geographic location of study ; (d)sample size; (e) study objective; (f) methodology; (g) school physical environmental variables; (h) health variables; (i) others variables and (j) main results. Missing data or definitions were resolved by direct author contact, where possible.

In addition, studies were divided into both children (6–12 years old) and adolescents (13–18 years old). If there are samples of both in the item, the item data will be located in both categories. (53)

Moreover, a second table will include the risk of bias assessment and the quality assessment.

29. * Analysis of subgroups or subsets.

State any planned investigation of 'subgroups'. Be clear and specific about which type of study or participant will be included in each group or covariate investigated. State the planned analytic approach.

After having the preliminary data, we will attend to analyze the data regarding different age groups, gender or other characteristic.

30. * Type and method of review.

Select the type of review, review method and health area from the lists below.

Type of review

Cost effectiveness

No

Diagnostic

No

Epidemiologic

No

Individual patient data (IPD) meta-analysis

No

Intervention

No

Living systematic review

No

Meta-analysis

No

Methodology

No

Narrative synthesis

No

Network meta-analysis

No

Pre-clinical

No

Prevention

No

Prognostic

No

Prospective meta-analysis (PMA)

No

Review of reviews

No

Service delivery

No

Synthesis of qualitative studies

No

Systematic review

Yes

Other

No

Health area of the review

Alcohol/substance misuse/abuse

No

Blood and immune system

No

Cancer

No

Cardiovascular

Yes

Care of the elderly

No

Child health

Yes

Complementary therapies

No

COVID-19

No

Crime and justice

No

Dental
No

Digestive system
No

Ear, nose and throat
No

Education
No

Endocrine and metabolic disorders
No

Eye disorders
No

General interest
Yes

Genetics
No

Health inequalities/health equity
Yes

Infections and infestations
No

International development
No

Mental health and behavioural conditions
No

Musculoskeletal
No

Neurological
No

Nursing
No

Obstetrics and gynaecology
No

Oral health
No

Palliative care

No

Perioperative care
No

Physiotherapy
No

Pregnancy and childbirth
No

Public health (including social determinants of health)
Yes

Rehabilitation
No

Respiratory disorders
Yes

Service delivery
No

Skin disorders
No

Social care
No

Surgery
No

Tropical Medicine
No

Urological
No

Wounds, injuries and accidents
No

Violence and abuse
No

31. Language.

Select each language individually to add it to the list below, use the bin icon to remove any added in error.
English

There is not an English language summary

32. * Country.

Select the country in which the review is being carried out. For multi-national collaborations select all the countries involved.

Spain

33. Other registration details.

Name any other organisation where the systematic review title or protocol is registered (e.g. Campbell, or The Joanna Briggs Institute) together with any unique identification number assigned by them. If extracted data will be stored and made available through a repository such as the Systematic Review Data Repository (SRDR), details and a link should be included here. If none, leave blank.

34. Reference and/or URL for published protocol.

If the protocol for this review is published provide details (authors, title and journal details, preferably in Vancouver format)

Add web link to the published protocol.

Or, upload your published protocol here in pdf format. Note that the upload will be publicly accessible.

No I do not make this file publicly available until the review is complete

Please note that the information required in the PROSPERO registration form must be completed in full even if access to a protocol is given.

35. Dissemination plans.

Do you intend to publish the review on completion?

Yes

Give brief details of plans for communicating review findings.?

36. Keywords.

Give words or phrases that best describe the review. Separate keywords with a semicolon or new line. Keywords help PROSPERO users find your review (keywords do not appear in the public record but are included in searches). Be as specific and precise as possible. Avoid acronyms and abbreviations unless these are in wide use.

37. Details of any existing review of the same topic by the same authors.

If you are registering an update of an existing review give details of the earlier versions and include a full bibliographic reference, if available.

38. * Current review status.

Update review status when the review is completed and when it is published.New registrations must be ongoing so this field is not editable for initial submission.

Please provide anticipated publication date

Review_Ongoing

39. Any additional information.

Provide any other information relevant to the registration of this review.

40. Details of final report/publication(s) or preprints if available.

Leave empty until publication details are available OR you have a link to a preprint (NOTE: this field is not editable for initial submission). List authors, title and journal details preferably in Vancouver format.

Give the link to the published review or preprint.
