# Supplementary material for: A Systematic Review of the Bibliometrics and Methodological Research Used on Studies Focused on School Neighborhood Built Environment and the Physical Health of Children and Adolescents
Source: Children (Basel). 2025 Jul 17;12(7):943. doi: 10.3390/children12070943 (PMC12293611; doi:10.3390/children12070943)

Extra figures

Figure S1 Continents

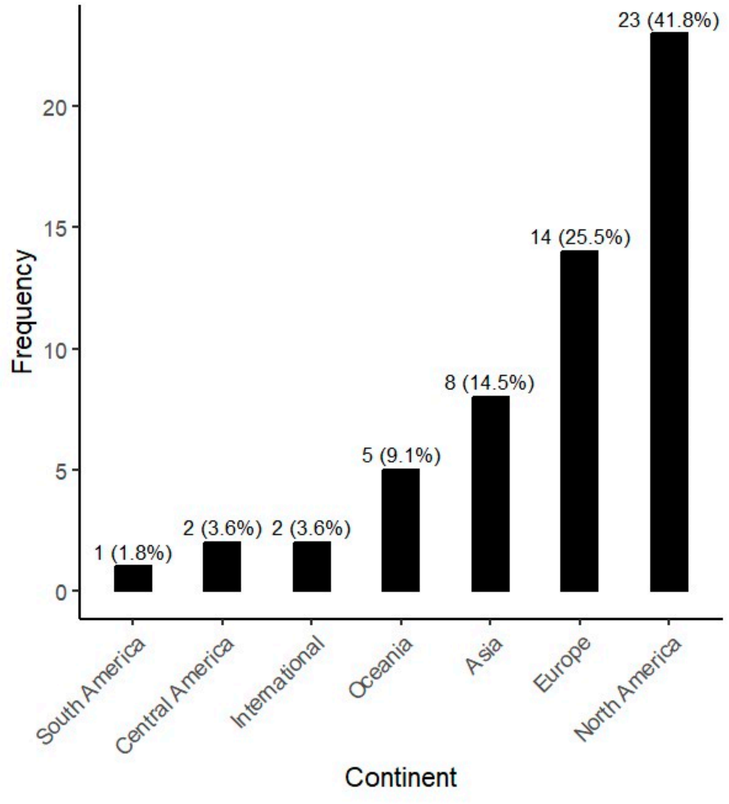

Figure S2 Study design

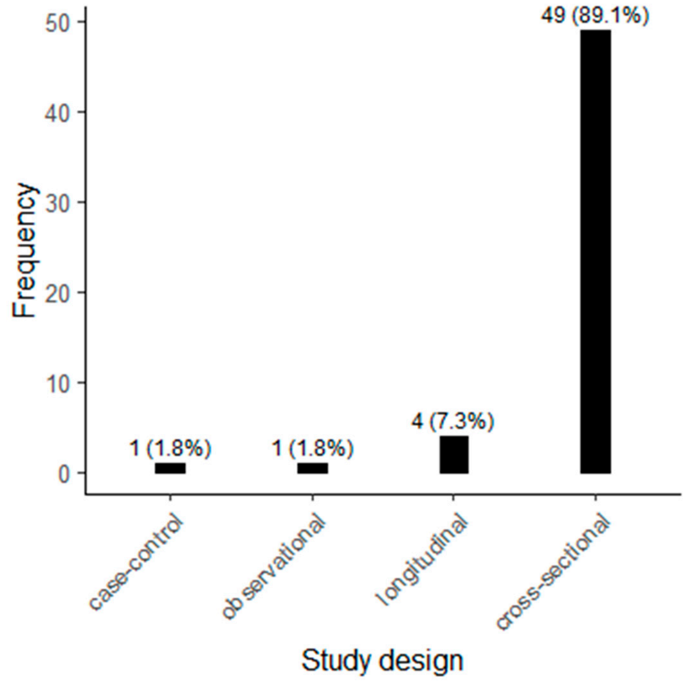

Figure S3 Setting

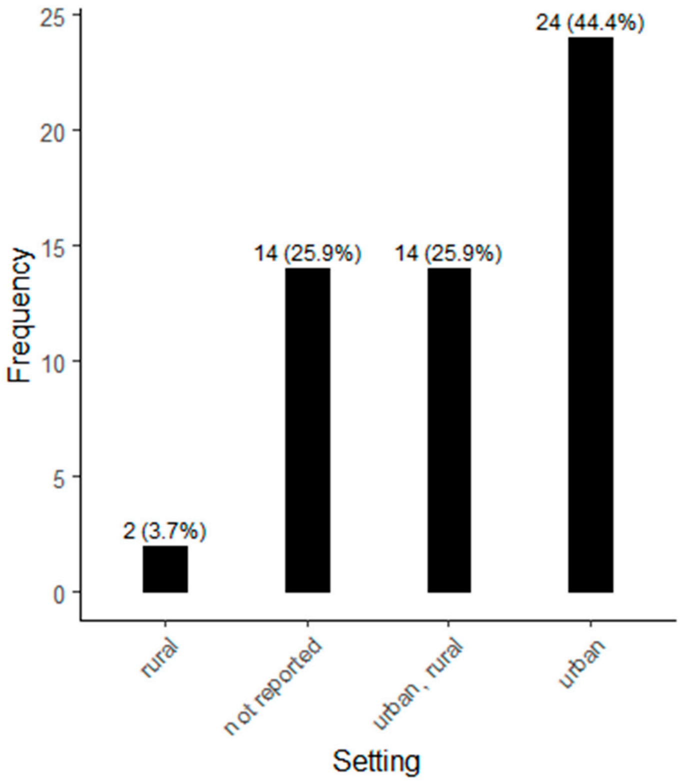

Figure S4 Participant's age

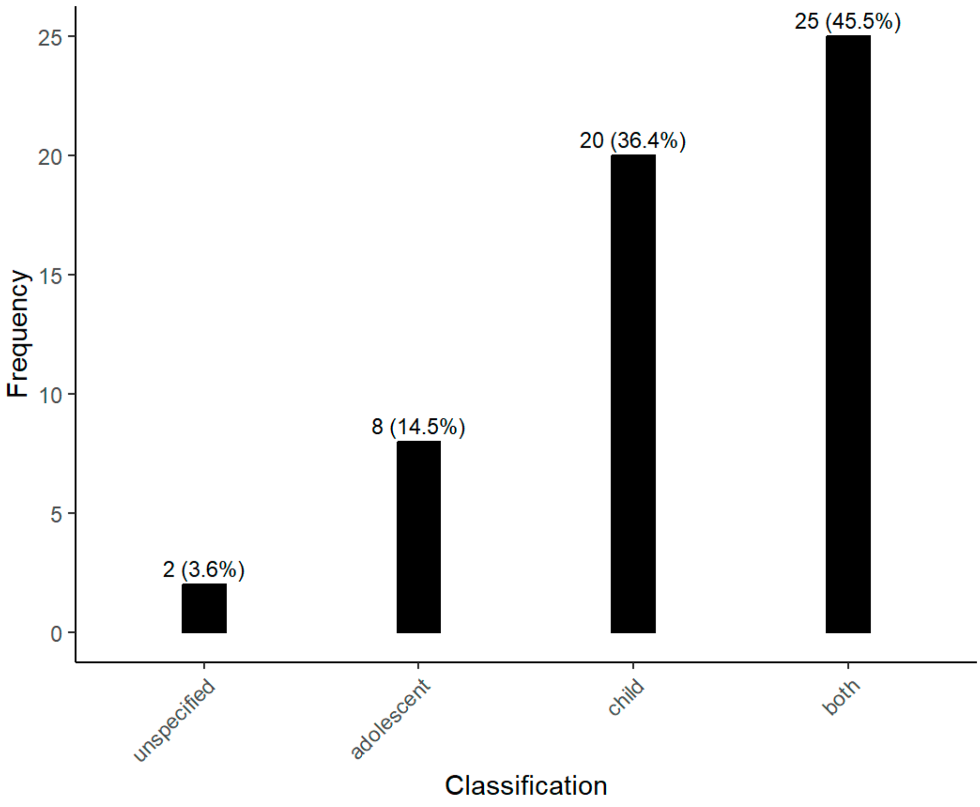

Figure S5 Female participants

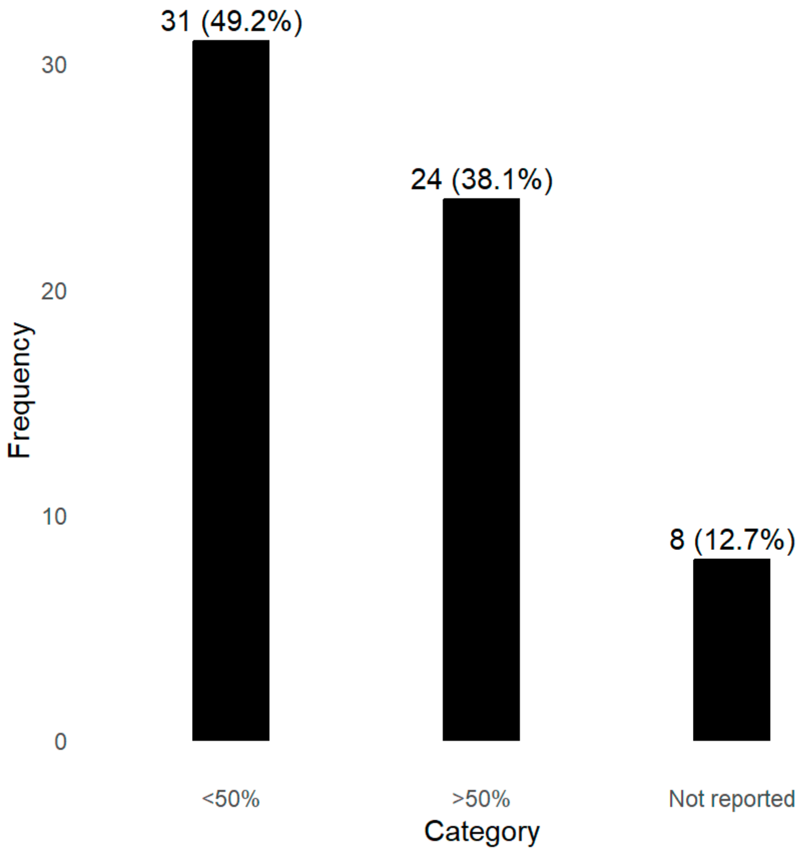

Figure S6 SNBE Variables

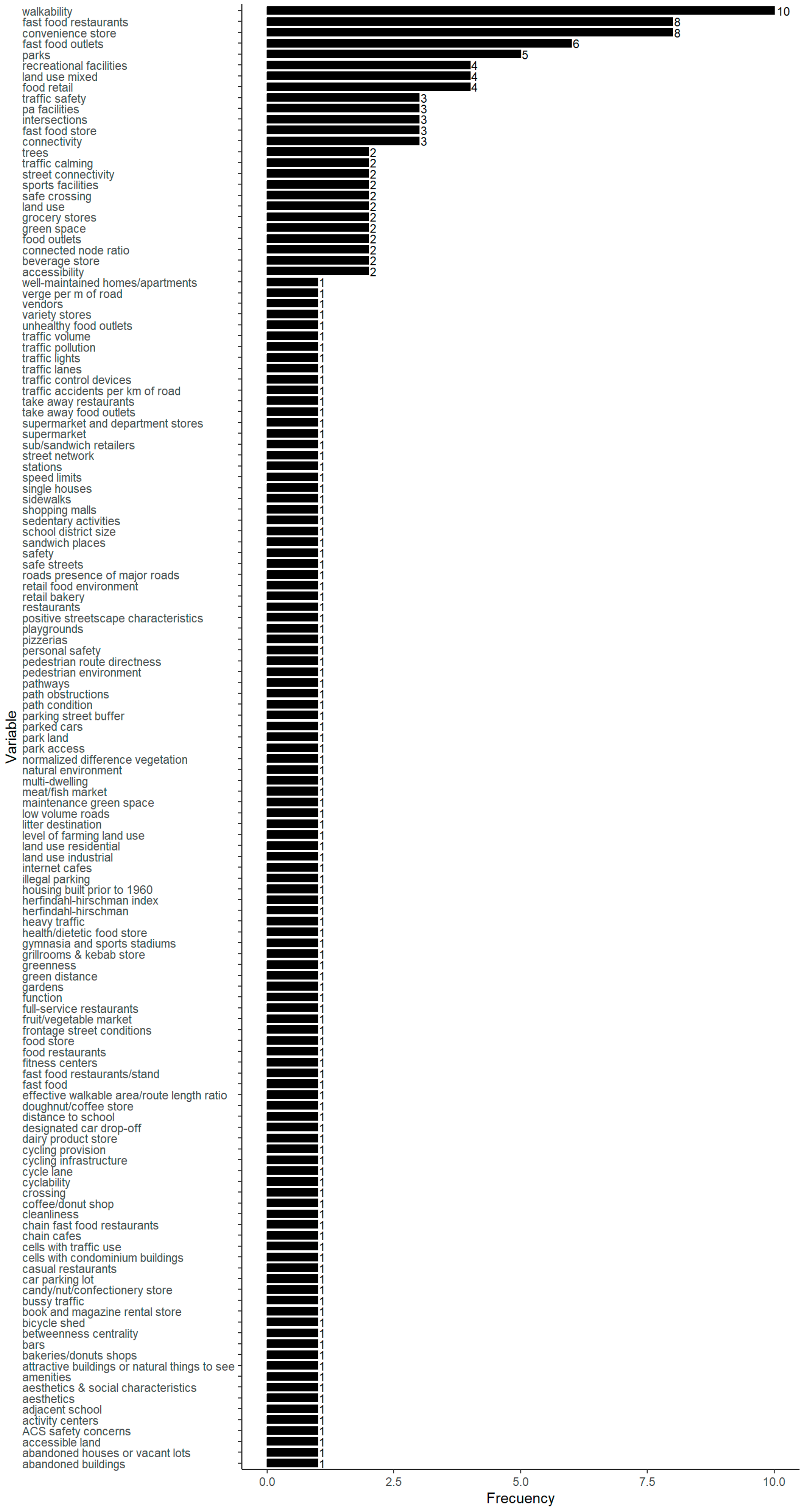

Figure S7 SNBE Specific Instrument

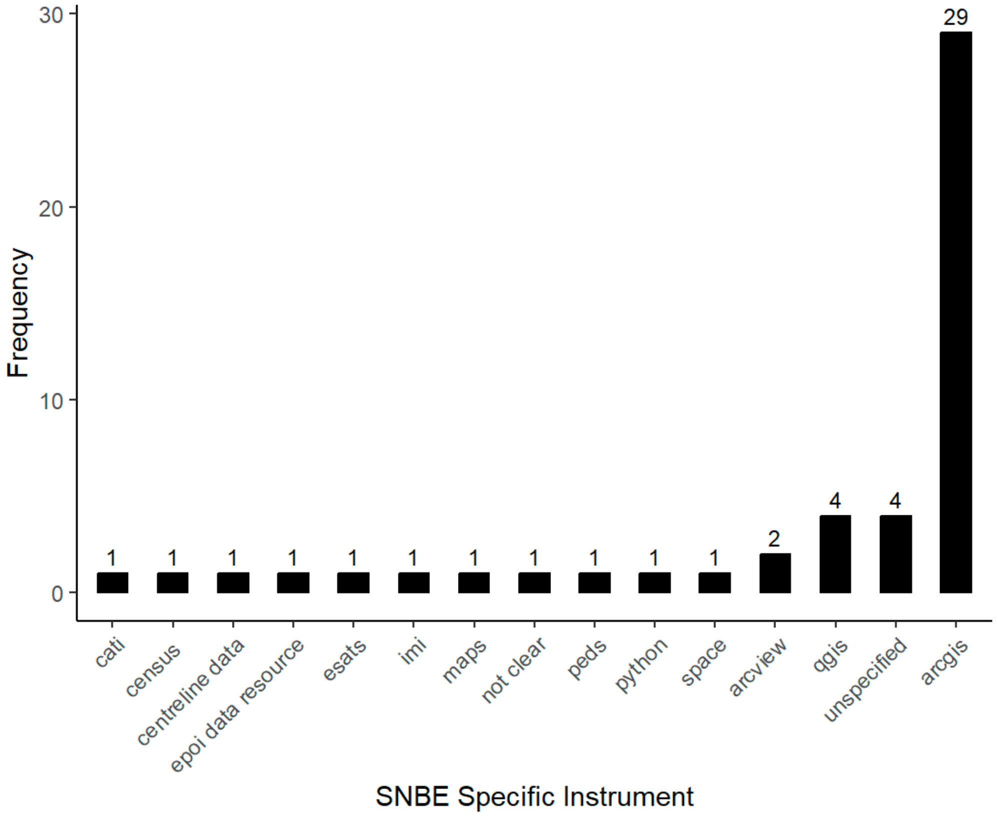

Figure S8 Instruments (PH)

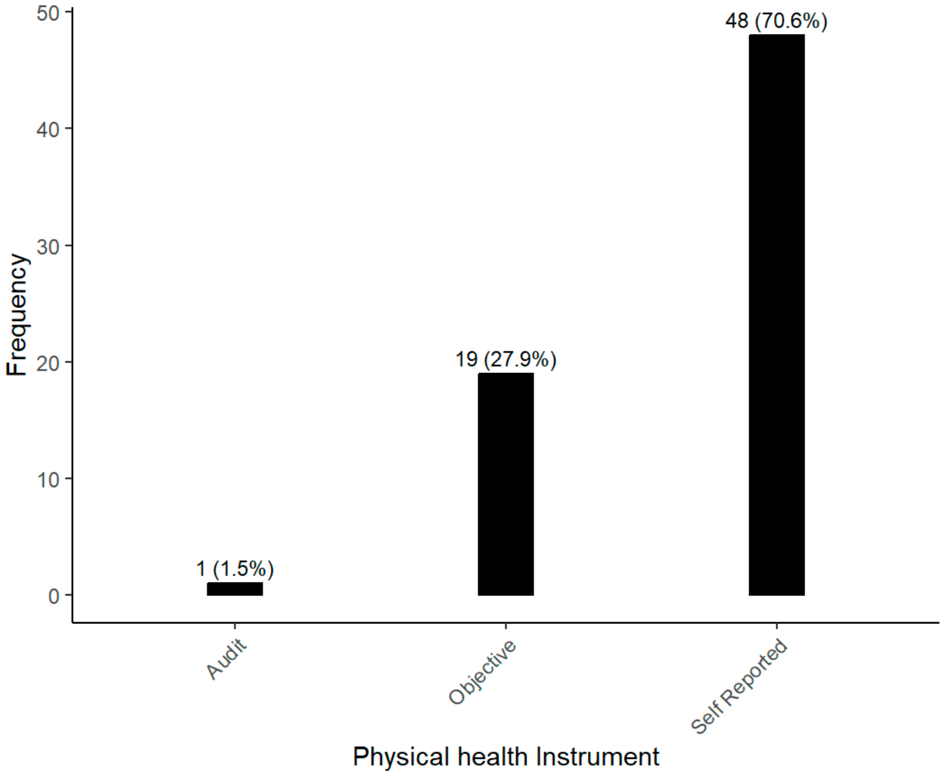

Supplement: Supplementary file 1 [file children-12-00943-s001.zip › Supplementary S3.pdf]
